# Early-stage sigmoid colon cancer resection followed by liver metastasis recurrence 1 year later and mesenteric recurrence more than 5 years later: a case report

**DOI:** 10.1186/s40792-023-01731-7

**Published:** 2023-08-31

**Authors:** Yumeto Mikuni, Michio Tani, Nobuki Ichikawa, Hiroki Matsui, Shin Emoto, Tadashi Yoshida, Takuya Otsuka, Shigenori Homma, Takahashi Norihiko, Akinobu Taketomi

**Affiliations:** 1 Department of Gastroenterological Surgery I, Hokkaido University, North 15 West 7, Kita-ku, Sapporo, Hokkaido 060-8638 Japan; 2 Department of Surgical Pathology, Hokkaido University Hospital, North 14 West 5, Kita-ku, Sapporo, Hokkaido 060-8648 Japan

**Keywords:** Colorectal cancer, Sigmoid colon cancer, Liver metastasis, Mesenteric recurrence, Early-stage, Distant period, Pathological indications, Additional resection

## Abstract

**Background:**

Early-stage colorectal cancer (CRC) is often treated endoscopically, but additional surgical resection may be considered depending on pathological findings.

**Case presentation:**

A 73-year-old man was found to have early-stage sigmoid colon cancer by colonoscopy during a medical examination, and endoscopic mucosal resection (EMR) was performed. The lesion was a 7-mm-sized sessile polyp, and the pathological diagnosis was well-differentiated tubular adenocarcinoma, pT1 (submucosal invasion of 400 μm), with no lymphovascular invasion, low budding grade, and negative horizontal and vertical margins. Therefore, the patient was observed without postoperative treatment. One year later, a computed tomography (CT) scan showed multiple liver metastases. After five courses of preoperative chemotherapy with folinic acid, 5-fluorouracil and oxaliplatin (FOLFOX) and panitumumab, liver metastases were reduced. The patient underwent extended right hepatic lobectomy. The pathological finding was well-to-moderately differentiated tubular adenocarcinoma, and immunohistochemistry findings were consistent with liver metastases from sigmoid colon cancer. Postoperatively, the patient received five courses adjuvant chemotherapy with FOLFOX. Although the patient had been recurrence-free for 5 years after liver resection, a CT scan revealed a nodular lesion in the sigmoid mesentery. Positron emission tomography (PET) showed abnormal accumulation in the same lesion. Therefore, the mesenteric nodules diagnosed as lymph metastasis and recurrence of sigmoid colon cancer and performed laparoscopic sigmoid colon resection with lymph node dissection. The pathological findings showed that the recurrent lesion in the mesentery formed a nodular infiltrate with venous, lymphatic, and neural invasion, but lymph node structures were not found, and it was assumed to be metastasis or recurrence due to lymphovascular invasion. The pathologic specimen of the sigmoid colon had no neoplastic lesions, which are considered to be a local recurrence on the mucosal surface. After sigmoid colectomy, adjuvant chemotherapy with CapeOX was conducted, and the patient has been recurrence-free for 13 months at present.

**Conclusion:**

Even early-stage CRCs that have no pathological indications for additional resection have risks of metastases and recurrences, and we may need to consider that the criteria for additional resection should not be limited to pathological findings alone.

## Background

Early-stage colorectal cancer (CRC) is often treated endoscopically, but additional surgical resection may be considered depending on pathological findings. Here, we report a case of a patient with early-stage CRC who did not meet the criteria for additional resection after endoscopic treatment but developed liver and mesenteric metastasis at a later date.

## Case presentation

**Fig. 1:**
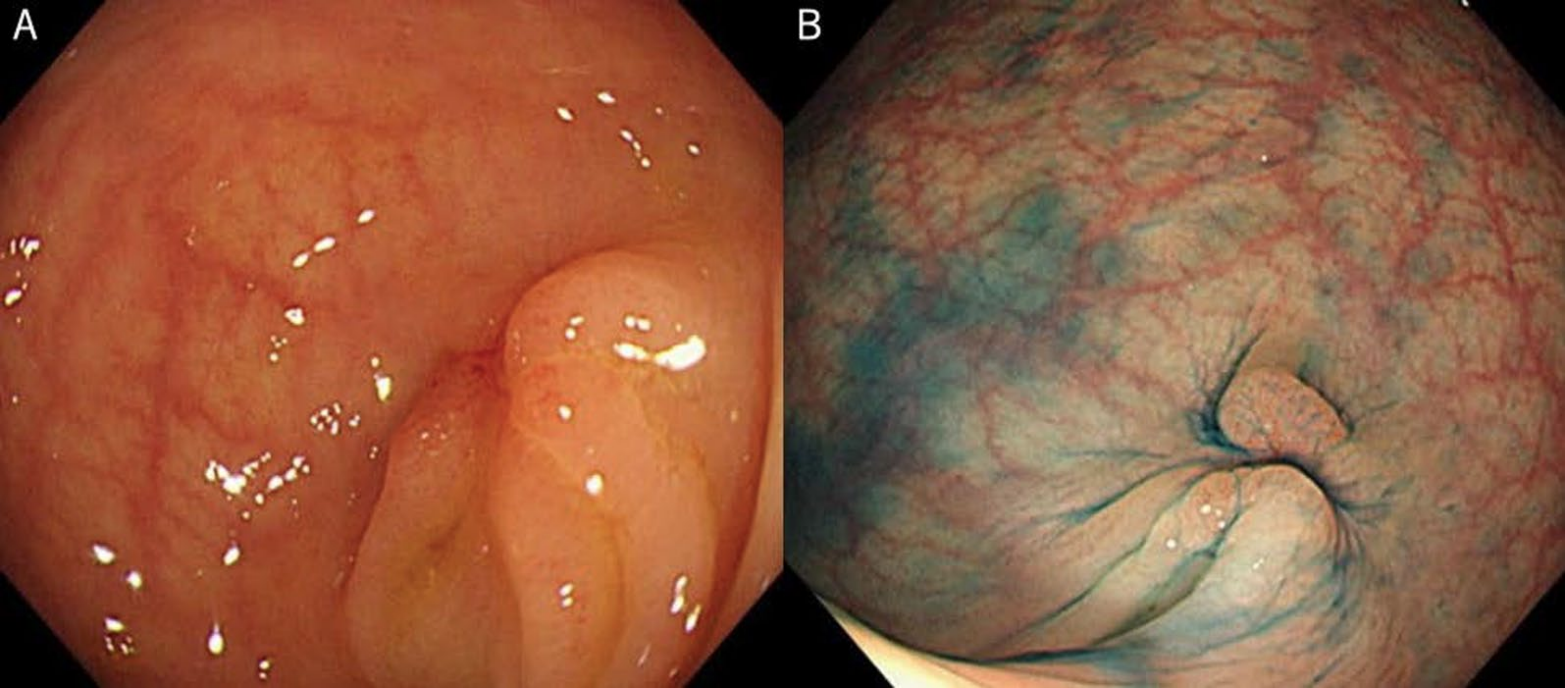
Colonoscopy revealed a 7-mm-sized sessile and protuberant lesion in the sigmoid colon. **A** Usual observation. **B** Observations after spraying indigo carmine

**Fig. 2:**
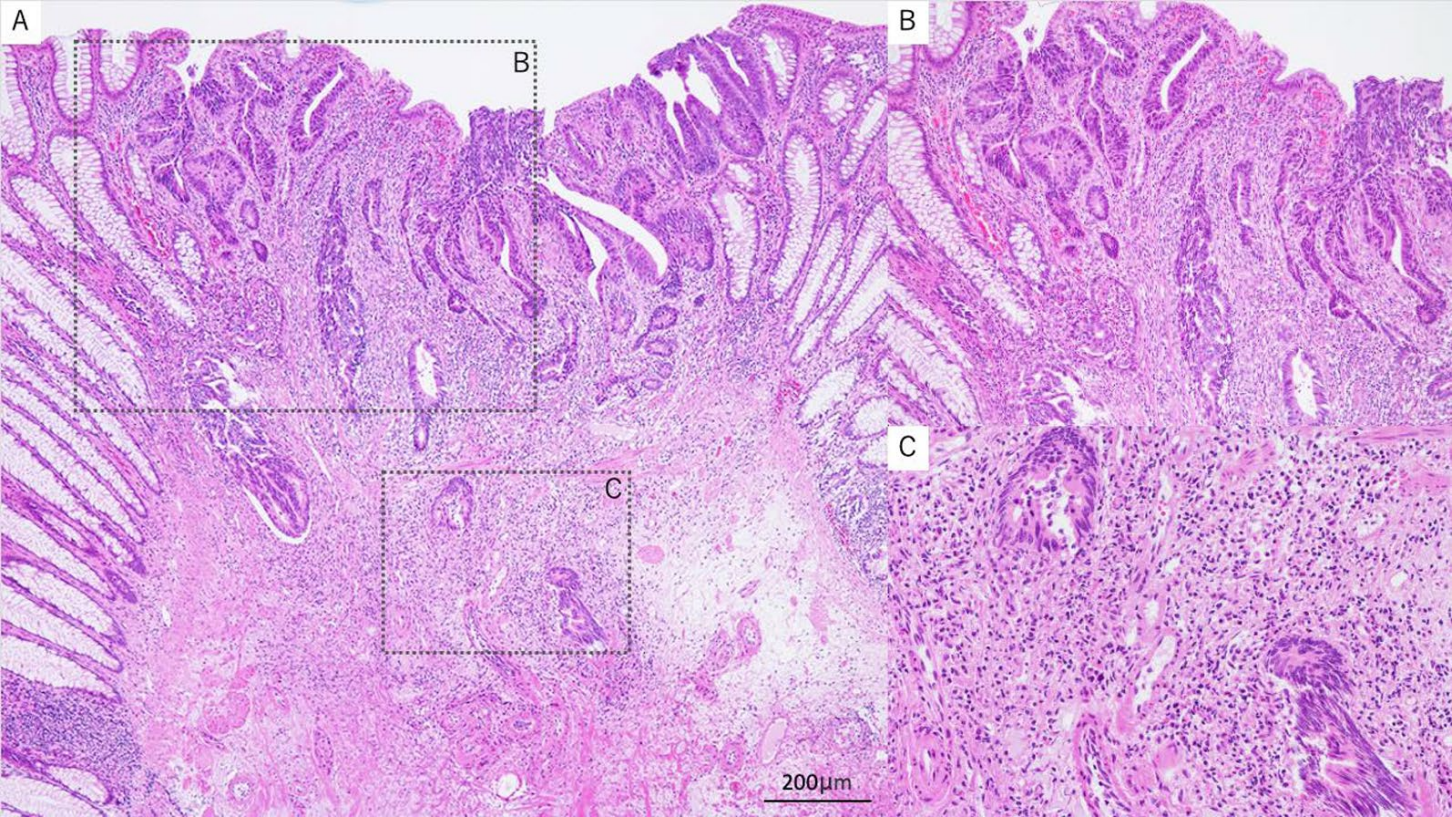
Pathological findings of EMR resection specimens (hematoxylin and eosin stain). **B** and **C** are enlargements of the dotted line in **A**. The deepest part of the tumor cells is located at C (SM400 µm). The magnifications are as follows (**A** ×40, **B** ×100, **C** ×200)

**Fig. 3:**
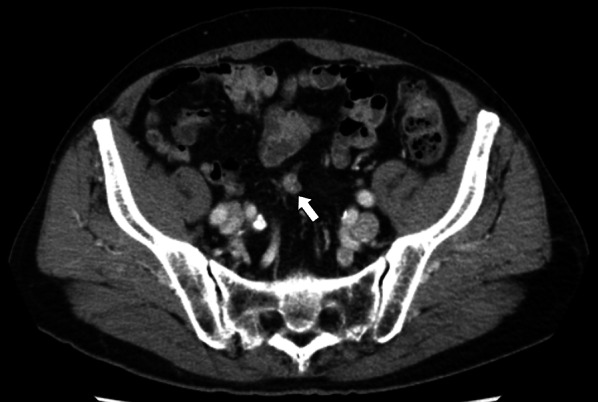
CT showed a nodular lesion in the sigmoid mesentery

**Fig. 4:**
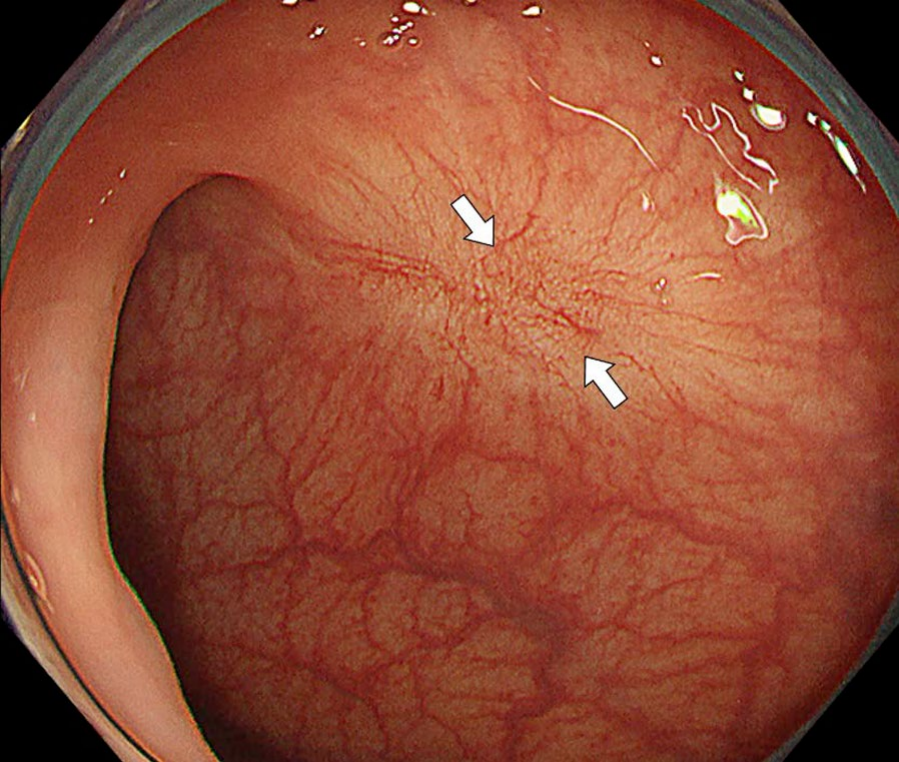
Lower gastrointestinal endoscopy showed only a post-EMR scar on the sigmoid colon mucosa

**Fig. 5:**
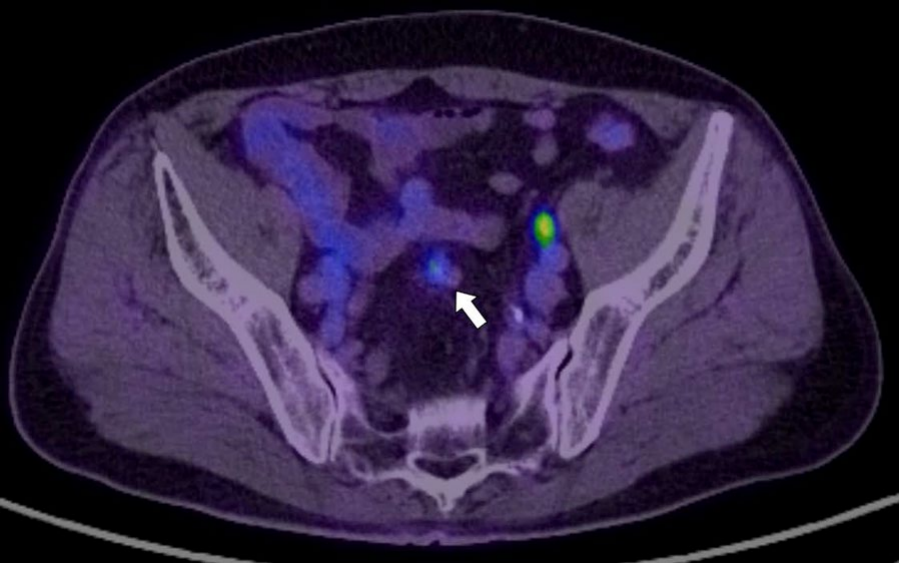
PET showed abnormal accumulation in the same lesion

**Fig. 6:**
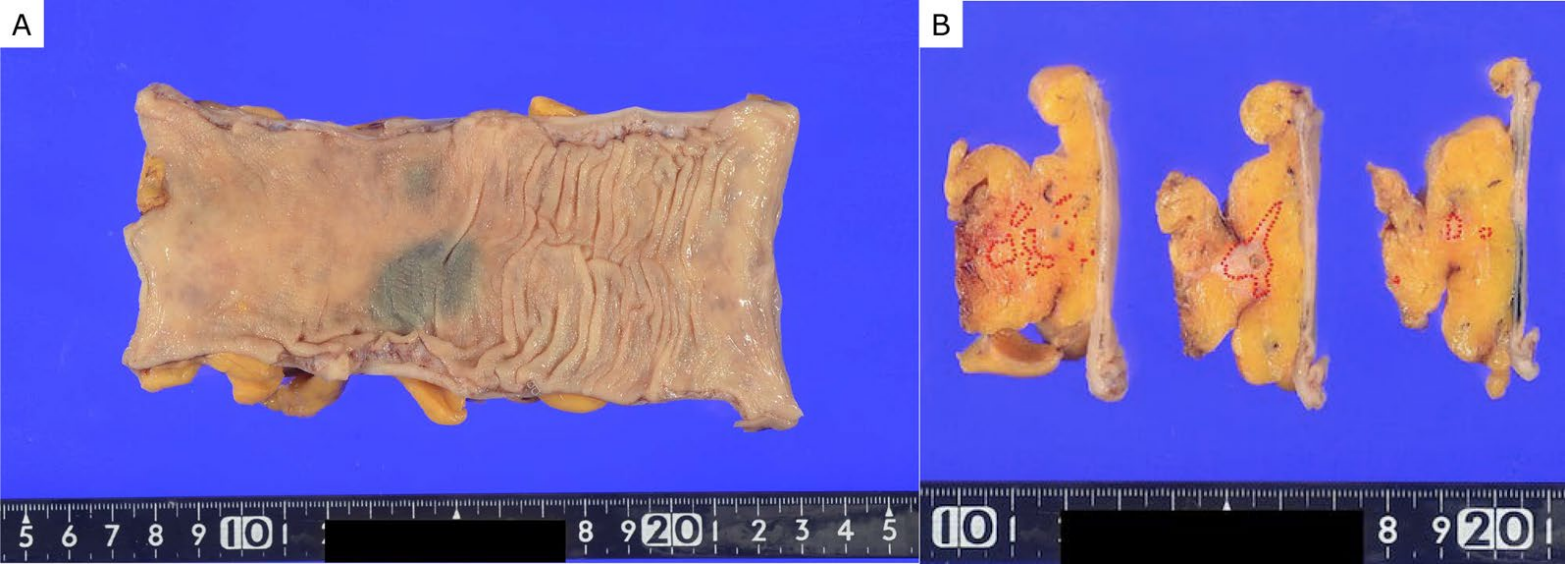
**A** Gross findings of resection specimen. **B** Mapping of tumor spread. (The area surrounded by the red line is adenocarcinoma.)

**Fig. 7:**
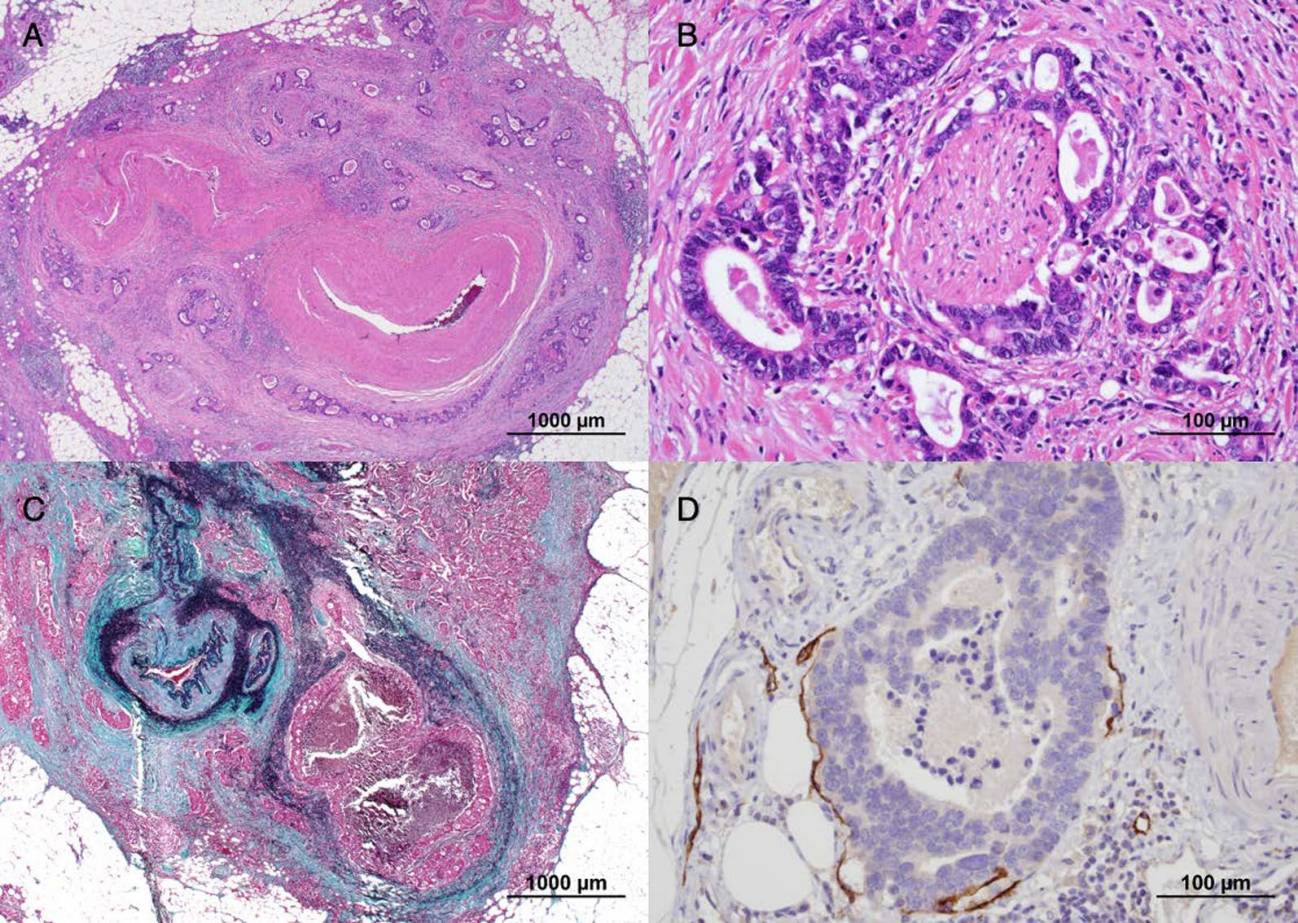
Pathological findings of the nodular lesions in the mesentery. The tumor showed a nodular-like distribution (**A** hematoxylin and eosin stain, ×20) with neural invasion (**B** hematoxylin and eosin stain, ×200), venous (**C** Elastica-Masson stain, ×20), and lymphatic invasion (**D** immunohistochemistry, D2–40, ×200)

A 73-year-old man was found to have early-stage sigmoid colon cancer by colonoscopy during a medical examination. Colonoscopy revealed a 7-mm-sized sessile and protuberant lesion in sigmoid colon; after observation with indigocarmine spray, we diagnosed early-stage sigmoid colon cancer (Fig. 1), and endoscopic mucosal resection (EMR) was performed. The pathological diagnosis was well-differentiated tubular adenocarcinoma, pT1 [submucosal (SM) invasion of 400 µm], with no lymphovascular invasion, low budding grade, and negative horizontal and vertical margins (Fig. 2). Both RAS and BRAF gene mutations were negative. Therefore, the patient was observed without postoperative treatment. CT scan 6 months after EMR showed no local recurrence, lymph node metastasis, or distant metastasis. One year later, a computed tomography scan showed multiple liver metastases of 35 × 45 mm in S4 and 30 × 35 mm in S8. Serum carcinoembryonic antigen (CEA) was elevated at 5.5 µg/ml (normal range < 5.0 µg/ml). We clinically diagnosed hepatic metastases from sigmoid colon cancer, and after five courses of preoperative chemotherapy with folinic acid, 5-fluorouracil and oxaliplatin (FOLFOX) and panitumumab, the liver metastases were reduced to 12 × 18 mm in S4 and 18 × 23 mm in S8. We evaluated this as a partial response, and the patient underwent extended right hepatectomy. The pathological finding was well to moderately differentiated tubular adenocarcinoma, and immunohistochemistry showed that tumor cells were weakly positive for cytokeratin (CK) 7 and positive for CK20 and Caudal-type homeobox 2 (CDX2), consistent with liver metastases from sigmoid colon cancer. The margins were evaluated to be negative. The tumors in S4 and S8 of the liver showed degeneration and necrosis in 1/3 and 2/3 of the tumors, respectively. Postoperatively, the patient received five courses of as FOLFOX adjuvant chemotherapy. Although the patient had been recurrence-free, a CT scan revealed a nodular lesion in the sigmoid mesentery 5 years after hepatectomy (Fig. 3). The serum CEA level was normal. Lower gastrointestinal endoscopy showed only a post-EMR scar on the sigmoid colon mucosa (Fig. 4), but positron emission tomography showed abnormal accumulation in the same lesion (Fig. 5). Therefore, we diagnosed lymph metastasis and recurrence of sigmoid colon cancer and performed laparoscopic sigmoid colon resection with lymph node dissection. Microscopically, the tumor was tubular adenocarcinoma which was compatible with recurrence of sigmoid colon cancer 7 years ago. Although the pathological examination revealed nodular-like distribution in mesentery with venous, lymphatic, and neural invasion, it did not contain lymph node metastasis (Figs. 6, 7). The pathologic specimen of the sigmoid colon had fibrosis in the submucosa, which was consistent with post-EMR scarring. No neoplastic lesions, which are considered to be a local recurrence on the mucosal surface, were found. After sigmoid colon resection, adjuvant chemotherapy with oxaliplatin and capecitabine was conducted, and the patient has been recurrence-free for 13 months at present.

## Discussion

Early-stage CRCs are divided into intramucosal (Tis) carcinomas and submucosal (T1) carcinomas. The overall recurrence rate is approximately 1% for SM carcinomas in Stage I cases [1]. Lymph node metastasis occurs in 6.8–17.8% [2–4] and liver metastasis occurs in 2.5% of submucosal invasive (T1) carcinomas [1]. Pathological endpoints to consider additional resections after EMR include SM invasiveness > 1000 µm [5], lymphovascular invasion [6, 7], poorly differentiated or signet cell mucinous carcinoma, and many buddings [7]. In particular, venous invasion is considered to be a risk factor for liver metastasis, and in a study of surgically resected cases of early-stage colorectal cancer (CRC), it was reported that venous invasion was significantly more frequent in cases with liver metastasis than in those without [8, 9]. Another study reported that skip lymphovascular invasion is associated with liver metastasis in patients with CRC [10]. Conversely, if pathological findings do not meet all of these criteria, the risk of metastasis or recurrence may be lower. The relapse ratio in pT1 carcinomas without any factors of additional resection has been reported to be 0% (*n* = 104) in patients with CRC [11]. In the present case, in spite of none of the pathological findings met the above criteria, the patient’s liver metastasis and mesenteric metastasis at distant stages were very rare. In fact, this is the first case report in which liver metastasis appeared after complete resection of early-stage colon cancer by EMR and mesenteric metastasis recurred at a distant period more than 5 years after hepatic resection.

Regarding recurrence at a distant period, recurrence over 5 years after radical resection for early-stage CRC is extremely rare, and lymph node recurrence after 5 years from the last surgery for stage I CRC is estimated to be less than 0.08% of all cases after curative resections [1]. One concept that may explain the validity of this case is tumor dormancy. Metastases and recurrences may arise from residual disseminated tumor cells [12]. Such metastases can occur several years after the primary treatment, because residual tumor cells can enter a dormant state and thus escape treatment [12]. In our case, the recurrence mechanism is interpreted as a late recurrence of residual skip lesions after a period of dormancy, which is consistent with early liver metastases, where skip lesions were present.

The course of the current case suggests that there are lesions that escape from chemotherapy without being completely obliterated and that they may be the cause of late recurrence. In other words, chemotherapy has limitations. Although the pathological diagnosis of our case did not meet the criteria for additional resection, the aforementioned reports [8–10] suggest a high possibility of hidden vascular invasion, and we retrospectively consider that we had an option to select simultaneous primary resection at the time the liver metastasis occurred.

In addition, in this case, although there were no pathological risk factors for surgical treatment just after EMR for primary resection, liver and mesenteric metastasis still occurred. Although we cannot completely rule out the possibility that the finding of lymphovascular invasion was overlooked, despite multiple pathological examinations, we found no evidence of lymphovascular invasion. In other words, subsequent surgical treatments could have been avoided if residual tumor cells could have been predicted and additional sigmoid colon resection could have been performed after EMR or at the same time as liver resection. Therefore, we need methods other than pathological factors that can correctly evaluate the risk of metastasis. The tumor marker CEA may be used as a complement for risk assessment but has limited specificity and sensitivity [13]. Recently, circulating tumor DNA (ctDNA) has attracted the attention of surgeons as a factor to assess the likelihood of recurrence other than pathological findings. Detection of ctDNA may signal the presence of minimal residual disease even in the absence of any other clinical evidence of disease [14]. Tie et al. showed that ctDNA was superior to standard clinicopathologic characteristics as a prognostic marker [15]. However, at present, there are no reports that verify this in cases limited to stage I early-stage CRC. Therefore, as more cases accumulate in the future, necessary and sufficient surgical indications may be identified when the indications for additional resection become more precise.

We have experienced a case of liver metastasis and late mesenteric recurrence after EMR for early-stage sigmoid colon cancer. It is necessary to keep in mind that such a course can occur even in patients with curative early-stage cancer after EMR.

## Conclusions

We reported a rare case of early-stage sigmoid cancer that caused liver metastasis and mesenteric metastasis at a distant stage occurring 7 years after the first surgery. Even early-stage CRCs that have no pathological findings for additional resection have risks of metastases and recurrences, and we may need to consider that the criteria for additional resection should not be limited to pathological findings alone.

## Data Availability

Not applicable.

## Abbreviations

CRC: Colorectal cancer
EMR: Endoscopic mucosal resection
SM: Submucosal
CT: Computed tomography
PET: Positron emission tomography
CEA: Carcinoembryonic antigen
FOLFOX: Folinic acid, 5-fluorouracil and oxaliplatin
CK: Cytokeratin
CDX2: Caudal-type homeobox 2
ctDNA: Circulating tumor DNA
